# 712. Neurocysticercosis at a Large Academic Center in the USA

**DOI:** 10.1093/ofid/ofab466.909

**Published:** 2021-12-04

**Authors:** Maya Ramanathan, Leopoldo Cordova, Jovanna Bertran-Lopez, Paola Lichtenberger, Paola Lichtenberger

**Affiliations:** 1 University of Miami Miller School of Medicine, Miami, FL; 2 University of Miami/Jackson Memorial Hospital, Miami, FL

## Abstract

**Background:**

Neurocysticercosis (NCC) is a parasitic infection that results from the ingestion of eggs from the adult tapeworm Taenia solium that develops when cysticercoids migrate into the central nervous system. In addition, this infection has been found to affect over 50 million individuals worldwide. In the United States, NCC mainly affects immigrants from Latin America, where the disease is endemic with seroprevalence rates ranging from 5% to 11%. Most data regarding NCC in the United States comes from hospital reports from California and Texas. We are undertaking this study to determine the differences seen in a higher Latin American and Haitian population compared to a previously seen predominantly Mexican population. In this retrospective review, we characterized the population diagnosed with NCC at one large tertiary medical center in South Florida, University of Miami Hospital.

**Methods:**

This retrospective chart review included adult patients from January 2009 to December 2019 with the admission or discharge diagnosis of neurocysticercosis (ICD 10 Code B 69.0 Neurocysticercosis and CPT code 86682 Cysticercosis). We extracted data on demographics, clinical symptoms, recurrence, treatment, resolution and follow up.

**Results:**

Forty-seven patients were analyzed to completion. Most of the cases were seen in Hispanics 72.3 % and from Central America 40.4%. The most common symptom was headache 53.2% followed by seizures 42.6%. Normal physical exam was noted in 93.6% of the cases. Most of the cases have 1-10 lesions (98%), located in the brain parenchyma (75%). Serum serology, CSF antibody or stool studies were not obtained in around 90% of the cases. Treatment was indicated in 70.2% of cases and recurrence was low at 17.0%. Refer to Tables 1-5 for full results.

Figure 1. Demographics and Clinical Symptoms

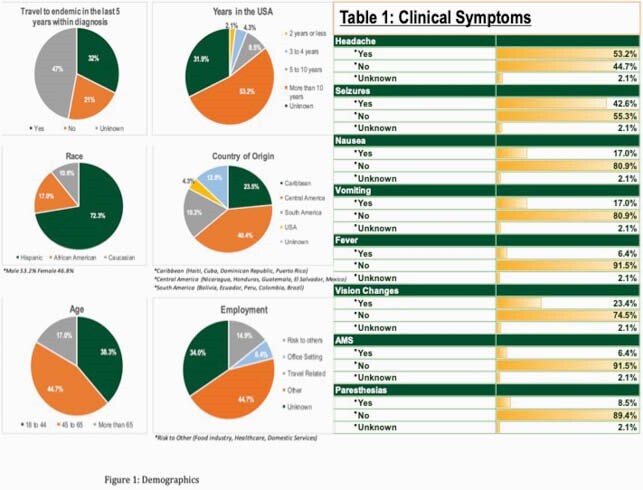

Figure 2. History and Imaging

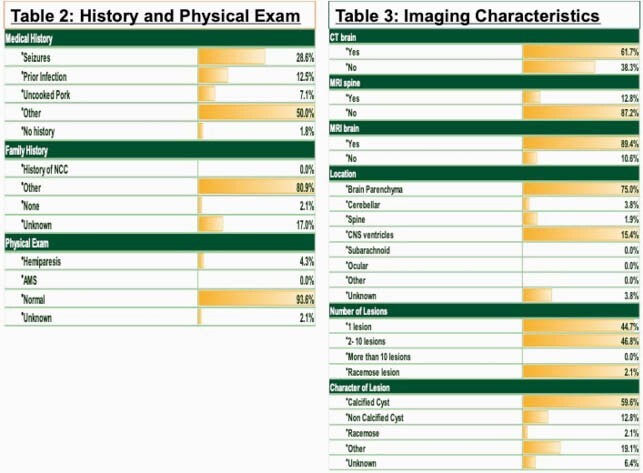

Figure 3. Laboratory Evidence and Follow up

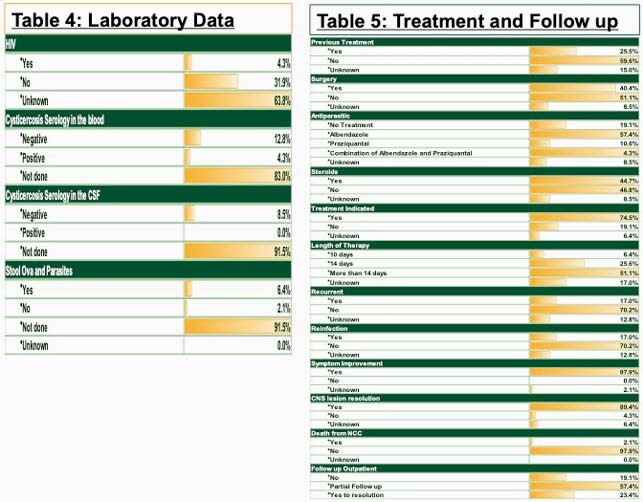

**Conclusion:**

NCC is a neglected tropical disease which is preventable. Our study noted that the majority of the affected population were immigrants that had been in the US for more than 10 years and came from central America and the Caribbean. With appropriate treatment, most of the symptoms and CNS lesions resolved, with a low mortality. Public health efforts to identify and treat the tapeworm carrier could be improved to allow for public health follow-up of cases. Although not yet considered endemic in Florida, we hope to bring awareness in this state.

**Disclosures:**

**All Authors**: No reported disclosures

